# Insights into the role of neuronal glucokinase

**DOI:** 10.1152/ajpendo.00034.2016

**Published:** 2016-05-17

**Authors:** Ivan De Backer, Sufyan S. Hussain, Stephen R. Bloom, James V. Gardiner

**Affiliations:** Section of Investigative Medicine, Division of Diabetes, Endocrinology and Metabolism, Imperial College London, London, United Kingdom

**Keywords:** glucokinase, glucose sensing, glucose homeostasis, appetite, counterregulatory response, neuronal

## Abstract

Glucokinase is a key component of the neuronal glucose-sensing mechanism and is expressed in brain regions that control a range of homeostatic processes. In this review, we detail recently identified roles for neuronal glucokinase in glucose homeostasis and counterregulatory responses to hypoglycemia and in regulating appetite. We describe clinical implications from these advances in our knowledge, especially for developing novel treatments for diabetes and obesity. Further research required to extend our knowledge and help our efforts to tackle the diabetes and obesity epidemics is suggested.

## BACKGROUND: GLUCOKINASE FUNCTION AND EXPRESSION

### Glucose-Sensing Neurons

glucose is a primary fuel source for the central nervous system (CNS) and is important for normal neuronal function (8). Neuronal glucose-sensing mechanisms allow the brain to constantly monitor neuronal glucose levels to control peripheral metabolic functions involved in energy and glucose homeostasis (87).

Glucose acts as a signaling molecule as well as an energy substrate in glucose-sensitive neurons. Two types exist: glucose-excited (GE) and glucose-inhibited (GI) neurons. Both GE and GI neurons are typically found in glucose-sensing brain regions such as the hypothalamus or brainstem (Table 1) (25, 73, 74, 167). The firing rate of GE neurons increases and that of GI neurons decreases as ambient glucose levels rise (42). Current evidence suggests that the majority of GE neurons express anorexigenic peptides, whereas GI neurons release appetite-stimulating peptides during hypoglycemic states to increase feeding (66, 109).

**Table 1.** The role of glucokinase in different brain regions

Brain Region	Role of Glucokinase	Mechanism	Type of Neuron	Ref. Nos.
ARC	Appetite, particularly for glucose-rich foods; counterregulatory response to hypoglycemia	K_ATP_ channels NPY; GHRH? Vagus; Reward?	GI	15, 20, 32, 42–44, 47, 58, 63, 70, 73, 75, 77, 79, 86, 92, 96, 113, 121, 142, 144, 145, 153
LH	Appetite: glucoprivic feeding	Orexin? Reward? Vagus?	GI	38, 56, 74, 77, 86, 92, 94, 113, 142, 147, 153, 171, 176
VMH	Glucose homeostasis	K_ATP_ channels; GABA; NO; vagus; adrenergic receptors	GE	16, 17, 23, 30, 40, 42, 50, 51, 58, 63, 73, 75, 81, 82, 88, 98, 99, 116, 141, 154, 155, 165, 172, 177
	Counterregulatory response to hypoglycemia			
MAN	Glucose homeostasis; counterregulatory response to hypoglycemia	Vagus?	?	41, 162, 174, 175,
AP	Energy homeostasis	?	?	170, 52, 3, 36
NTS	Glucose homeostasis	K_ATP_ channels?	?	2, 37, 42, 84, 92, 158, 173
		GLUT2?		
DMV	Glucose homeostasis	K_ATP_ channels?	?	2, 92, 126

GI, glucose inhibited; GE, glucose excited; GHRH, growth hormone-releasing hormone; ARC, arcuate nucleus; LH, lateral hypothalamus; VMH, ventromedial hypothalamus; MAN, medial amygdalar nucleus; AP, area postrema; NTS, nucleus tractus solitarius; DMV, dorsal motor nucleus of the vagus.

### Glucokinase in the Periphery

Glucokinase, also known as hexokinase IV, catalyses the conversion of glucose to glucose-6-phosphate, which constitutes the first step of glycolysis. In most cells, it is catalyzed by hexokinase I. Glucokinase has certain biochemical properties that differentiate it from other hexokinases and allow it to function as a glucose-sensing enzyme (89). It has a lower affinity for glucose than other hexokinases (*K*_m_ ∼10 mmol/l) and is not saturated at physiological glucose concentrations. Unlike other hexokinases, glucokinase is not inhibited by the product of the reaction it catalyzes. These properties allow the rate of glucose phosphorylation to be dependent on and proportional to intracellular glucose levels (95).

Glucokinase is expressed in the liver and pancreas (68, 159). It exists as two different isoforms with the same kinetic properties but different functions (67). These isoforms are encoded by the same gene, but separate promoters lead to different splicing patterns, producing different variants of the glucokinase enzyme (135). The function of glucokinase in the pancreas is well established. Pancreatic glucokinase is involved in the process of glucose-stimulated insulin secretion (GSIS). It plays a key role in sensing alterations in glucose levels and triggering insulin release. A rise in glucose concentration results in increased cellular adenosine triphosphate (ATP) production, causing the closure of ATP-sensitive potassium (K_ATP_) channels and the depolarization of the β-cell. Calcium (Ca^2+^) influx through voltage-gated Ca^2+^ channels ensues (73, 89, 130), leading to insulin release. In the liver, glucokinase has a central role in promoting the uptake of glucose and its subsequent conversion to glycogen for energy storage (45, 114, 130, 159). Mutations in the glucokinase gene lead to abnormalities in glucose homeostasis in rodents and humans, whereas abnormalities in glucokinase function in the pancreas and liver have been implicated in diabetes mellitus (13, 117).

### Neuronal Glucokinase

**Fig. 1.**
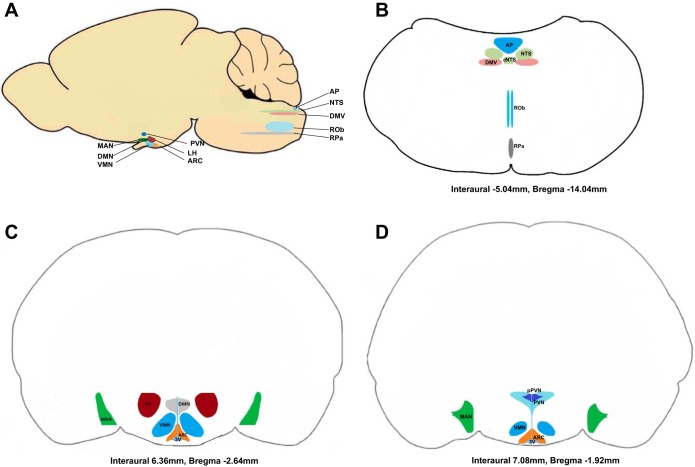
Location of main brain centers containing glucokinase-expressing neurons in the rat brain. *A*: sagittal section diagram illustrating the position of the brain regions in the rat brain-expressing glucokinase believed to be involved in glucose sensing, which are located mostly in the hypothalamus and in the brainstem. *B*: coronal section diagram of glucokinase-expressing nuclei of the brainstem. *C*: coronal section diagram of glucokinase-expressing hypothalamic nuclei. *D*: coronal section diagram of glucokinase-expressing nuclei closer to the forebrain. MAN, medial amygdalar nucleus; PVN, paraventricular nucleus; pPVN, parvocellular PVN; LH, lateral hypothalamus; VMN, ventromedial nucleus; DMN, dorsomedial nucleus; ARC, arcuate nucleus; AP, area postrema; NTS, nucleus tractus solitarius; DMV, dorsal motor nucleus of the vagus; ROb, raphe obscurus; RPa, raphe pallidus; LV, lateral ventricle; chp, choroid plexus; 3V, third ventricle; d3V, dorsal 3rd ventricle. cNTS, central nucleus tractus solitarius.

The expression of glucokinase mRNA and protein has been demonstrated in multiple neuronal populations in the CNS in rats (Fig. 1), mice, and humans (2, 22, 42, 59, 90, 92, 93, 134, 135). Glucokinase is expressed in numerous hypothalamic nuclei, including the arcuate nucleus (ARC), ventromedial nucleus (VMN), and lateral hypothalamic area (LHA) (90, 92, 111). Glucokinase mRNA has also been detected in the paraventricular nucleus (PVN) and dorsomedial nucleus (DMN), although very little is known about its function in these nuclei. Outside of the hypothalamus glucokinase has been identified in the medial amygdalar nucleus (MAN) (92). It was also found in the three nuclei that make up the dorsal vagal complex (DVC) of the brainstem, the *nucleus tractus solitarius* (NTS), the area postrema (AP), and the dorsal motor nucleus of the vagus (DMV). All DVC nuclei play an important part in regulating homeostatic processes (Fig. 1) (42, 90). Glucokinase is also expressed in glial cells such as hypothalamic tanycytes (46, 139). Glucokinase in these cells is thought to have an important role in energy homeostasis; however, this review will focus on neuronal glucokinase.

**Fig. 2.**
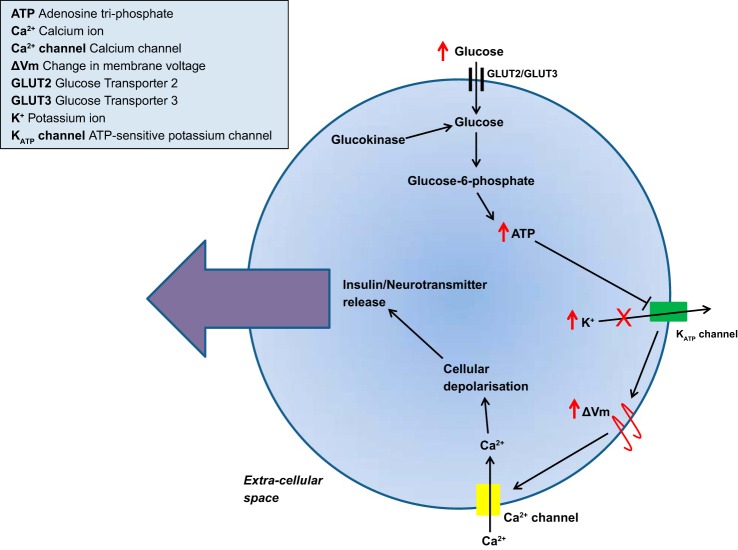
Role of glucokinase in the peptide release mechanism of pancreatic β-cells and glucose-excited neurons. Glucokinase activity leads to cellular depolarization, followed by insulin secretion in pancreatic β-cells or neurotransmitter release in glucose-excited neurons. As extracellular glucose concentrations increase, glucose is taken up into the islet cell predominantly by glucose transporter 2 (GLUT2) (158) and into the neuron predominantly via GLUT3 glucose transporters (160). Once in the cytosolic space, glucose is phosphorylated by glucokinase to form glucose 6-phosphate (95). Although this reaction consumes adenosine triphosphate (ATP), the levels of ATP ultimately rise due to further glycolysis of glucose. The coupling of glucose entry with glycolysis and ATP production allows the increase in ATP concentration to inhibit ATP-sensitive potassium (K_ATP_) channels. This prevents the efflux of K^+^ ions. As a result K^+^ ions accumulate within the neuron, and the membrane potential of the cell rises. The difference in membrane voltage triggers the influx of Ca^2+^ ions through voltage-gated Ca^2+^ channels. Ca^2+^ entry causes cellular depolarization, which in turn leads to an action potential (130). This proposed mechanism allows glucokinase to function as a glucose sensor by coupling glucose availability with β-cell and neuronal activity and insulin and neurotransmitter release (108).

Neuronal glucokinase mRNA has a splicing pattern similar to the pancreatic isoform, suggesting that it has a similar role to the pancreatic isoform (135, 139). The neuronal form of the enzyme is thought to play a central role in glucose sensing in GE neurons (6, 69, 73) via a mechanism comparable with that of glucokinase in pancreatic β-cells (Fig. 2) (42). In keeping with this, the involvement of K_ATP_ channels in neuronal glucose sensing (12, 123) and colocalization of glucokinase and K_ATP_ channels has been demonstrated in several studies (92, 161).

Glucokinase plays a central role in both GE and GI glucose-sensing neurons (42, 73, 74). The glucose-sensing mechanism of GE neurons is similar to that of pancreatic β-cells. As glucose levels rise, glucose enters the neuronal cell via glucose transporter 2 (GLUT2). There it is phosphorylated by glucokinase, increasing the cytosolic ATP/ADP ratio and causing the closure of K^+^_ATP_ channels (12, 60, 89). Neuronal depolarization triggers Ca^2+^ ion entry via Ca^2+^ channels, leading to neurotransmitter secretion (Fig. 2) (42, 74).

**Fig. 3.**
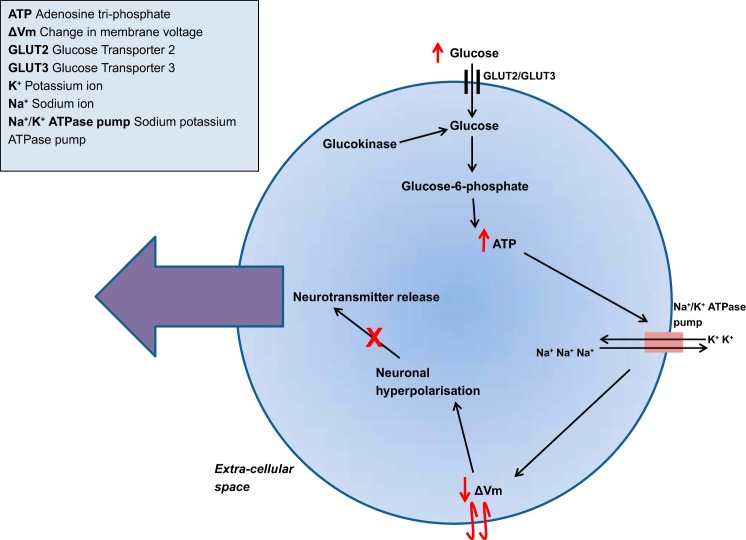
Proposed mechanism by which glucokinase activity leads to neuronal hyperpolarization and inhibits neurotransmitter release in glucose-inhibited neurons. As extracellular glucose concentrations increase, glucose is taken up into the islet cell predominantly by GLUT2 (158) and into the neuron predominantly via GLUT3 glucose transporters (160). Once in the cytosolic space, glucose is phosphorylated by glucokinase to form glucose 6-phosphate (95). Although this reaction consumes adenosine triphosphate (ATP), the levels of ATP ultimately rise due to further glycolysis of glucose. The coupling of glucose entry with glycolysis and ATP production allows the increase in ATP concentration to stimulate sodium potassium ATPase (Na^+^/K^+^ ATPase) pumps. For one ATP molecule, each pump pumps three Na^+^ ions out of the cell and enables the entry of two K^+^ ions. This causes a decrease in membrane voltage and results in hyperpolarization of the cell (80), ultimately leading to a decrease in neuronal firing.

The mechanism underlying glucose sensing in GI neurons is less well understood. Calcium imaging studies reveal that >70% of GI neurones in the VMN are affected by GK inhibitors (42, 73), suggesting that GK is involved in glucose sensing in GI neurons. Their activity is reduced in the presence of glucose due to hyperpolarization of the cell. The extent of GK involvement is unclear, although hyperpolarization has been proposed to occur via stimulation of Na^+^/K^+^ ATPase pumps caused by a glucokinase-induced rise of ATP levels within neurons, leading to inhibition of neuronal activity (Fig. 3) (80). An alternative, glucokinase-independent mechanism, has also been postulated. GI neurons may become hyperpolarized following glucose-induced activation of postsynaptic cystic fibrosis transmembrane regulator (CFTR) Cl^−^ channels (27, 151) via the activation of adenosine monophosphate-activated protein kinase (AMPK) and nitric oxide signaling (110, 151). Further studies are needed to shed light on this mechanism.

### Glucokinase-Independent Glucose Sensing

Neuronal glucose sensing does not rely entirely on glucokinase; non-glucokinase-dependent glucose-sensing mechanisms also exist. For instance, the cellular energy sensor AMPK is also involved in this process. In rats, VMN AMPK knockdown abolished the glucagon response to hypoglycemia, whereas pharmacological activation of AMPK in the VMN improved the response to hypoglycaemia (100, 101). AMPK is believed to enable ventromedial hypothalamic GI neurons to depolarize in response to decreased glucose levels via a mechanism involving nitrous oxide (NO) and cyclic guanosine monophosphate (cGMP) (110), with hyperglycemia having the opposite effect (29). Another important energy sensor, per-arnt-sim kinase (PASK), may also play a role in neuronal glucose sensing. Its expression varies acutely in accord with glucose levels, and it may be involved in the signaling mechanism of AMPK-mediated glucose sensing (61, 62). Glucose sensing via sodium-coupled glucose cotransporter (SGLT) 1–3 has also been reported (115). The mechanism by which signals from different metabolites are integrated to generate a net neuronal output effecting homeostasis and the complex interplay between neuronal sensors such as SGLTs, AMPK, and PASK still needs further investigation. It is also important to note that in glucokinase-expressing neurons, other hexokinases are present to produce ATP regardless of variations in extracellular glucose concentrations.

### The Role of Neuronal Glucokinase

The recent insights into the role of glucokinase in glucose-sensing neurons will be detailed in this review. It builds on previous work that provides a strong evidence base for its function in different brain regions and extends the importance of glucokinase beyond the hypothalamus. An understanding of this important neuronal metabolic sensor will undoubtedly help promote our understanding of disease processes and lead to effective drug development.

## GLUCOKINASE AND THE REGULATION OF GLUCOSE HOMEOSTASIS

The most clearly defined role of neuronal glucokinase is for the regulation of glucose homeostasis. This appears to be mediated mainly by glucokinase in the VMH and MAN through modulation of the counterregulatory response (CRR). Other mechanisms involving glucokinase in the DVC may be at play, but this remains to be demonstrated conclusively.

### Glucokinase and the Counterregulatory Response

Studies have shown that glucokinase in the ventromedial hypothalamus (VMH), VMN, and medial amygdalar nucleus plays a central role in the CRR, a feedback system to counteract hypoglycemia by increasing production of glucose and limiting its utilization (98). It is characterized by the release of glucagon, which suppresses the secretion of insulin and augments gluconeogenesis and glycogenolysis, catecholamines, and other hormones (4).

#### Glucokinase in the ventromedial hypothalamus: regulator of the counterregulatory response to hypoglycemia.

**Fig. 4.**
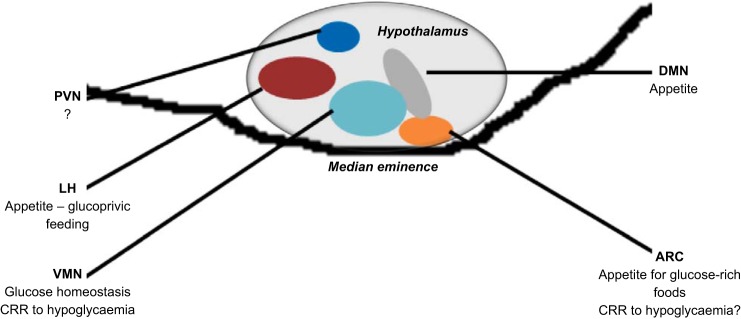
Postulated roles of glucokinase in the hypothalamus. Summary illustration describing the role of glucokinase in each of the major hypothalamic nuclei expressing the glucose sensor. PVN, paraventricular nucleus; LH, lateral hypothalamus; VMN, ventromedial nucleus; DMN, dorsomedial nucleus; ARC, arcuate nucleus; CRR, counterregulatory response.

The hypothalamus has long been described as an important center for the regulation of glucose homeostasis (157) as well as for appetite (32, 96). For more than 40 years, evidence has been generated demonstrating that it is a center for glucose-sensing (27, 43, 83, 97, 108, 138). Various regions of the hypothalamus express glucokinase, but to date glucokinase in the ventromedial hypothalamus (VMH) (73) has been the main focus of research (Fig. 4).

Intracarotid infusion of glucose increases c-fos expression in VMH neurons, a well-established marker of neuronal activation (58). Whereas insulin-induced hypoglycaemia (IIH) increased glucokinase expression and neuronal activity in the VMH (75), reduction of glucokinase mRNA by 90% in cultured VMH neurons using RNA interference abolished all demonstrable glucose-sensing ability (73). In a low-glucose environment, pharmacological activation of glucokinase increases neuronal activity in GE neurons and decreases that of GI neurons, as demonstrated by changes in Ca^2+^ oscillations (73). These findings suggest that plasma glucose levels alter neuronal activity via glucokinase in VMH neurons, with glucokinase being the glucose sensor. In support of this, electrophysiology studies have revealed that glucokinase inhibition decreases GE neuronal activity, whereas it increases that of GI neurons (42, 74, 167).

It is important to note that hexokinase I is also expressed in VMH glucose-sensing neurons (75). Hexokinase I has a much higher affinity for glucose (*K*_m_ <1 mmol/l). However, unlike glucokinase, its kinetic properties prevent it from modulating its activity according to ambient glucose concentrations (129). Therefore, in the VMH, hexokinase I appears to drive the metabolism of glucose to maintain a constant supply of ATP, regardless of fluctuations in extracellular glucose concentrations, whereas glucokinase acts as a glucose sensor by biochemically coupling glucose flux to cellular processes that may be distinct from cellular ATP production (11, 129).

McCrimmon (98) describes VMH glucokinase as having a pivotal role in inducing the CRR to hypoglycemia. This is backed by the findings of Sanders et al. (141), who reported that injections of the glucokinase inhibitor alloxan into the third ventricle impaired the CRR to hypoglycaemia in rats.

Initial studies have focused on the VMH as a whole rather than specifically examining the role of ARC and VMN glucokinase in the CRR. The VMN is regarded as an important hypothalamic glucose-sensing center, and glucokinase has been implicated as the primary glucose sensor (Fig. 4) (73). Indeed, hypoglycemia increased the sensitivity of glucose-sensing neurons in parallel with an increase in glucokinase mRNA within the VMN (88).

Stanley et al. (153) demonstrated colocalization of glucokinase and growth hormone-releasing hormone (GHRH) in ARC neurons. GHRH neurons mediate the secretion of growth hormone (GH) (153), which is released during hypoglycemia as part of the CRR (137). Although less important than immediate sympathetic nervous system responses such as glucagon and adrenaline release, GHRH release has been implicated in the generation of the CRR and is part of the later neurohormonal CRR cascade (10, 55, 164). Because glucokinase activity leads to neurotransmitter secretion in other neurons (63), it is possible that ARC glucokinase may induce GH release from GHRH-expressing neurons in response to a decrease in ambient glucose levels (51). A direct link between glucokinase activity and GH release has not been shown, however, and additional research is required to establish the role of glucokinase in GH secretion.

Recurrent hypoglycemia is known to blunt the CRR to subsequent hypoglycemic episodes (116, 118). Studies have shown that antecedent IIH increases glucokinase mRNA expression in the VMH (42, 75, 118). This upregulation could lead to a requirement for a lower glucose level in VMH glucose-sensing neurons to initiate the CRR by increasing glycolytic flux in the neurons regulating the CRR. Levin et al. (88) reported that in vivo microinjection of the glucokinase activator *compound A* diminished the CRR to acute hypoglycemia, whereas selective downregulation of VMH glucokinase had the opposite effect. Therefore, by having a pivotal role in glucose sensing, VMH glucokinase may act as regulator of the CRR to hypoglycemia. The presence of VMH glucokinase activity allows reductions in glucose to be sensed during hypoglycemia and is important for the initiation of the CRR. However, variations in the activity of glucokinase may alter the glucose threshold at which the CRR to hypoglycemia is initiated.

The mechanism behind the effects of VMH glucokinase on the CRR is unknown. Pharmacological activation of K_ATP_ channels in the VMH enhanced the CRR to hypoglycemia in rats (99). K_ATP_ channels thus seem to play a role in glucose-sensing neurons in the detection of hypoglycemia and in the generation of the CRR. Because they are expressed in glucokinase-expressing VMH neurons and are involved in the enzyme's downstream signaling pathway (12, 63), K_ATP_ channels may form part of the mechanism mediating hypothalamic glucokinase's effects on the pancreas. Supporting this, iVMH administration of the K_ATP_ channel blocker glibenclamide inhibited the secretion of glucagon and adrenaline in response to both systemic hypoglycemia and central glucopenia (48). This study suggests a link between the VMH and the pancreas, which has also been postulated in other studies. For instance, microinjection of the nonmetabolizable glucose analog 2-deoxyglucose into the VMH induced the release of glucagon, adrenaline, and noradrenaline, and this response was blocked by iVMH glucose infusion (23, 24). The CRR may be triggered by the inhibition of VMH GABAergic neurons following the decrease of hypothalamic glucose levels (16–17, 30, 177), suggesting that glucokinase in GE neurons mediates the CRR. Nitric oxide has also been implicated in the generation of the response, but not in GABAergic neurons (50). The VMH is likely to be linked to the periphery via sympathetic and parasympathetic connections, both of which innervate pancreatic α-cells (4). These connections could occur via the brainstem, which is known to relay hypothalamic autonomic signals to the gut (4). Sympathetic nerve stimulation resulted in glucagon secretion, and this response was abolished by the α-adrenergic receptor blocker phentolamine (81). Hence, the VMH may cause glucagon release through splanchnic sympathetic innervation of pancreatic α-cells, perhaps by releasing adrenaline and noradrenaline acting on α_2_- and β_2_-adrenergic receptors located on the α-cells (17, 31, 82, 154–156). Vagal cholinergic pathways, which form part of the parasympathetic nervous system, have also been implicated in the autonomic regulation of glucagon secretion, as muscarinic M3 receptor activation resulted in glucagon release (165). Acetylcholine may also act directly on adrenal cells to induce adrenaline release (116).

#### Glucokinase in the medial amygdalar nucleus contributes to initiation of the counterregulatory response.

The presence of glucose-sensing neurons in the MAN has been demonstrated as subcutaneous injections of 2-DG-increased c-Fos activity (41). A role in the CRR has been postulated as stimulation of the amygdala-increased glucagon secretion, whereas lesions had the opposite effect (71). Glucokinase is expressed in the MAN and may be responsible for the detection of hypoglycemia and the initiation of the CRR (175). Zhou et al. (175) report that lesions in the MAN suppressed whereas 2-DG infusion amplified the CRR/IIH in vivo. In addition, MAN glucoprivation during mild systemic hypoglycaemia amplified the CRR. However, local glucoprivation (caused by injection of 2-DG) in the MAN alone is insufficient to generate a counterregulatory hormone response, suggesting that MAN glucose sensing plays only a contributory role to other regions involved in CRR, such as the VMH.

The signaling pathways between the MAN and the gut are poorly understood. They may involve the vagus nerve, as studies have shown that the MAN projects directly to the NTS and DMV, which are known to relay signals from the forebrain to the gut via vagal efferents, although this has been shown mostly in the central nucleus of the amygdala rather than the MAN (162, 163, 174). The mechanism behind the effect of MAN glucokinase on the CRR has not been explored.

### Glucokinase in the Dorsal Vagal Complex: Glucose Sensor Roles but Uncertain Function

The DVC of the brainstem is a large structure containing the NTS, AP, and DMV, all of which possess glucose-sensing properties. It is considered to be an important center for glucose homeostasis and has been associated with the CRR (132, 157).

Each of the DVC nuclei expresses low levels of glucokinase (5, 42). IIH increased glucokinase mRNA expression in the DVC (53), whereas prolonged hyperglycemia in streptozotocin-induced diabetic rats decreased it (59). This suggests a role for glucokinase in glucose sensing. Whole cell patch clamp recordings testing the neuronal response to hypoglycemia showed that glucokinase is expressed in both GE and GI neurons (14); however, further evidence supporting this claim is sparse, and additional research is needed to determine its exact role in the DVC.

#### Glucokinase in the area postrema.

The AP has an incomplete blood-brain barrier (BBB), which enables glucose to diffuse into the DVC (170). Therefore, it may play a role in glucose sensing in this region. Glucose-sensing properties have been discovered in the AP (2, 131), and glucose-sensitive neurons of the AP were shown to be both stimulated and inhibited by varying concentrations of glucose (1, 52). Due to its glucose-sensing properties, the AP may be involved in glucose homeostasis (3).

Contreras et al. (36) suggested a role for the AP in energy homeostasis, as lesions to this brain region induced hypophagia and rapid body weight loss in rats. They described the possibility of a complementary role between the AP and the NTS in the regulation of caloric intake potentially mediated by glucokinase. However, no work has been conducted to confirm these authors' claims, and the role of glucokinase in the AP remains unclear.

#### Glucokinase in the nucleus tractus solitarius.

The existence of glucose-sensitive neurons in the NTS has been demonstrated (2, 173). For instance, a glucoprivic feeding response induced by the administration of 2-deoxyglucose and 5-thioglucose to the fourth ventricle was lost following lesioning of the NTS, suggesting that this nucleus possesses glucose-sensing properties (104, 133, 149). Whole cell and on-cell patch clamp recordings have demonstrated both increased and decreased neuronal excitability in the NTS in response to an elevation in environmental glucose, suggesting the presence of both GE and GI neurons (25). Evidence supports the presence of GLUT2 and K_ATP_ channels (37, 158), two glucose-sensing components, in the NTS. GLUT2 neurons have been linked with the CRR, as they are activated by hypoglycaemia and contribute to glucagon secretion (84).

Reverse transcription-polymerase chain reaction (RT-PCR) suggests low expression levels of glucokinase in the NTS (42). In situ hybridization studies did not detect its presence within this nucleus, however (92), perhaps because in situ hybridization may not be sensitive enough to detect the low levels of glucokinase present in the NTS. Very little research has been done to link glucokinase to the glucose-sensing machinery of the NTS, and its role in this nucleus remains unexplored.

#### Glucokinase in the dorsal motor nucleus of the vagus.

The DMV contains glucose-sensitive neurons (2). Along with the NTS, it may also be part of a hypothalamus-brainstem-liver circuit regulating liver gluconeogenesis. Pocai and colleagues (125, 126) have suggested that hypothalamic lipid sensing triggers a signal to the NTS, possibly via the activation of K_ATP_ channels. It is then transferred to the DMV, which relays it to the liver via a vagal efferent pathway. The DMV thus appears to play a role in the regulation of peripheral glucose homeostasis.

Low levels of glucokinase are expressed in the DMV (92); however, its physiological role in this nucleus has not been investigated. So far, no evidence has implicated it in the DMV's glucose-sensing mechanism.

## GLUCOKINASE AND THE REGULATION OF APPETITE

The relationship between glucose sensing and feeding behavior has been postulated following earlier work demonstrating the colocalization of the receptor for the anorexigenic hormone GLP-1, glucokinase, and glucose transporters in brain areas controlling feeding behavior and containing glucose-sensing neurons (6).

Although for some time direct evidence has been lacking, recent data provide strong evidence for the role of glucokinase and neuronal glucose sensing in appetite. Hypoglycemic and euglycemic clamps have shown an influence of glucose levels on appetite, particularly for high-calorie foods (121). Rodent data have highlighted a role for hypothalamic glucose sensing in appetite regulation (63). In humans, differential patterns of neuronal activation can result from changes in glucose concentrations and alter food-seeking behaviour (121). Increasing evidence linking glucokinase in the ARC and LH to the regulation of appetite will be discussed in this section.

### Glucokinase in the Arcuate Nucleus: Taste-Independent Promoter of Glucose-Rich Foods

Depletion of VMH glucokinase did not change spontaneous feeding, body weight, or glucose tolerance, but it caused a reduction in glucoprivic feeding, thus suggesting a role for VMH glucokinase in this process (43).

The VMH contains the ARC, a nucleus that has been implicated in the regulation of appetite since it contains both the orexigenic neuropeptide Y (NPY) and agouti-related peptide (AgRP) neurons and anorexigenic proopiomelanocortin (POMC) and cocaine- and amphetamine-regulated transcript neurons (35, 57, 72).

Glucokinase is expressed at relatively high levels in the ARC (42, 92, 113) and in POMC and NPY neurons (66, 109, 123). The medial ARC is adjacent to the median eminence. The capillaries of this circumventricular organ form fenestrations during times of low glucose availability (106, 124) to enable the movement of glucose from the bloodstream into the ARC to maintain a steady nutrient supply when diffusion of glucose via GLUT1, GLUT3, and GLUT4 transporters present in the BBB (112) is not sufficient. The ARC appears to be an important glucose-sensing center, as it modulates glucose entry depending on plasma glucose concentrations.

Guillod-Maximin et al. (58) showed that an intracarotid injection of glucose triggered a significant increase in the number of Fos-positive nuclei, indicative of neuronal activation, in the ARC compared with rats injected with vehicle, suggesting that increasing plasma glucose activates ARC neurons. Alterations in peripheral glucose levels modulate glucokinase expression. Indeed, fasting, a state associated with lower glucose levels and increased motivation to consume food, increases ARC glucokinase levels (63, 75, 142). Conversely, ARC glucokinase activity decreases in streptozotocin-induced diabetic rats, presumably because of the prolonged hyperglycemia (113). Therefore, ARC glucokinase may be expressed in GI neurons and play a role in appetite regulation by allowing the ARC to respond to changes in glucose and alter appetite.

We have recently found evidence suggesting that glucokinase in the ARC regulates energy homeostasis by using pharmacological and genetic approaches to specifically increase glucokinase activity in the ARC of rodents (63). Upregulated ARC glucokinase activity increased chow intake and specifically increased glucose intake Wistar in rats. Conversely, a reduction in ARC glucokinase activity decreased consumption of these foods. Interestingly, only glucose consumption was affected when both glucose and chow were available. Fructose intake remained unchanged (63), suggesting that ARC glucokinase controls glucose appetite via a taste-independent mechanism possibly analogous to that in *Drosophila* (44). That work further provided a mechanism involving K_ATP_ channels and changes in glucose-stimulated NPY release in mediating the effects of glucokinase. Previous works support the involvement of glucokinase in NPY release as well as the colocalization of glucokinase and K_ATP_ in NPY-containing neurons (92, 161). Alterations in NPY expression have also been shown following the manipulation of K_ATP_ channels (78, 122).

The mechanism leading to increased glucose intake following glucokinase activation may include the PVN and DMN. ARC NPY neurons project to the parvocellular division of the PVN (pPVN) and to the DMN. These projections were shown to influence carbohydrate intake, as a positive correlation was shown between carbohydrate ingestion and NPY levels in the ARC, pPVN, and DMN (70, 79). NPY may stimulate feeding by activating Y1 and Y5 receptors in these hypothalamic nuclei (63). The pPVN may in turn relay the signal to the brainstem, leading to the release of orexigenic peptides in the gut via vagal efferents through a well-known forebrain-hindbrain-gut pathway (20, 144). However, this pathway is more commonly associated with satiety signaling (18–22). Another possible mechanism involves the paraventricular thalamic nucleus (PVT). ARC NPY neurons project to the PVT, possibly via the LHA (47, 77). A study showed that PVT neurons receiving NPY terminals from the ARC provide divergent axon collaterals to the nucleus accumbens (NA) (86), suggesting that the promoting effects of ARC glucokinase on glucose intake may be driven by reward signals. However, this seems unlikely, as the intake of fructose, which is more associated with a hedonic response (145), was unaffected by changes in glucokinase expression (63). Further investigation is required to shed light on the mechanism responsible for the effects of ARC glucokinase on the appetite for glucose-rich foods.

Several studies do not support a role for ARC glucokinase in appetite regulation. VMH knockdown of glucokinase activity with alloxan and short-hairpin RNA, delivered via an adenoviral vector, did not change appetite regulation at 24 h and 14 days, respectively (43). Limitations of this study included use of a cytotoxic glucose analog (140) and immune responses in relation to adenoviral gene transfer (76). Intracerebroventricular (icv) administration of a nonspecific glucokinase inhibitor, glucosamine, increased glucoprivic feeding and stimulated hypothalamic NPY secretion, which directly contradicts our findings (176). In addition, Levin et al. (88) observed no changes in food intake when VMH glucokinase activity was pharmacologically altered. However, these studies did not target the ARC exclusively, so the pharmacological agents in the hypothalamus may have had confounding effects.

### Glucokinase in the Lateral Hypothalamic Area: Mediation of Glucoprivic Feeding?

The glucose-sensing center of the LH is traditionally divided into two sections, the lateral hypothalamic area (LHA) and the perifornical area. Both of these areas possess glucose-sensing properties since increases in peripheral glucose concentrations induced c-fos immunoreactivity (153), and orexin-expressing neurons within them are activated by hypoglycemia (26, 28, 105).

Glucokinase mRNA is moderately expressed in the LHA of rats (92, 113) and rainbow trout (127). Conversely, it has not been detected in neurons of the perifornical area. Glucose sensing in this area is likely to utilize a mechanism not involving glucokinase, and thus the rest of this section will focus on the LHA.

The glucose-sensing role of LHA neurons was demonstrated when cell firing was altered by varying glucose concentrations. One study demonstrated the number of action potentials of the glucose-sensitive neurons (forming ∼30% of LHA cells) increased following a reduction in glucose concentration. Raising glucose levels had the opposite effect, suggesting that LHA glucose-responsive cells are GI in type. Silver and Erecinska (147) proposed a “glucokinase-type” enzyme as a possible mechanism for the detection of changes in glucose concentration. A different study demonstrated changes in the levels of Ca^2+^ intracellular LHA neurons in response to glucose (107). These studies support the work of Stanley et al. (153), who used intraperitoneal 2-deoxy-d-glucose (2-DG) injections to mimic hypoglycemia to assess neuronal activation in glucokinase-expressing cells stained with yellow fluorescent protein. 2-DG induced a significant increase in c-fos immunoreactivity in yellow fluorescent protein-immunopositive neurons in the LH (153). Although unspecified by the authors, immunoreactivity is likely to have occurred in the LHA, as glucokinase is not expressed in the perifornical area. The c-fos response to hypoglycemia suggests that glucokinase-expressing cells in this area play a role in the neural pathways activated by low glucose.

LHA glucokinase may play a role in glucose-sensing, as its activity in rat LH slices decreased upon exposure to increasing glucose concentrations (142). Moreover, its activity in the LHA was increased during IIH (33), suggesting that glucokinase may be expressed in GI neurons. Zhou et al. (176) investigated its role via icv injections of its inhibitor glucosamine. They reported that glucosamine stimulated feeding and induced c-fos activation in the LHA. Glucosamine-stimulated c-fos was detected within the appetite-stimulating orexin neurons. Hence, glucokinase within this neuronal population may play a role in the sensing of hypoglycemia and mediating of glucoprivic feeding.

LHA glucokinase may induce glucoprivic feeding by stimulating a hedonic response, implying a role in food reward. A high density of orexinergic neuronal projections from the LH terminates in the PVT, which acts as a relay center to the NA (86, 94). This circuit could be powered by cholinergic interneurons via muscarinic receptor activation to convey information regarding energy balance to output neurons and may involve enkephalin, among other peptides (77). The majority of orexigenic projections to the PVT originate in the perifornical area of the LH rather than the glucokinase-expressing LHA (86); however, orexinergic connections between the LHA and the PVT also exist (94). LHA glucokinase may also mediate glucoprivic feeding through direct connections between the LHA and the gut, as diet restriction induced neuronal activation of the LHA in mice (143). This brain-gut connection appears to occur via the vagus nerve and may be bidirectional, as vagotomy impaired LHA neuronal activation induced by intragastric infusions of various glutamate-containing solutions (38, 171).

Others dispute the role of glucokinase in glucose sensing within LHA orexin neurons. They demonstrated that orexin cell glucose sensing is not affected by glucokinase inhibitors and presented evidence that glucokinase is not expressed in orexin neurons (56). Evidence supporting the involvement of glucokinase in LHA glucose sensing is controversial, and additional investigation is required to determine its function in this region.

## GLUCOKINASE IN OTHER CNS REGIONS

The presence of glucose-sensing neurons in parvocellular neurons of the PVN has been demonstrated (102). Glucokinase mRNA has been detected in the PVN as well as its regulatory protein (7), although its role in this hypothalamic nucleus has not been clearly identified. Glucokinase expression is found in oxytocin and vasopressin neurons of the supraoptic nucleus located in the PVN, where it has been postulated as a glucose sensor. Indeed, increases in glucose stimulated oxytocin and vasopressin release during a hypothalamic explant study in a glucokinase-dependent manner. It also increased cellular Ca^2+^ levels (148, 150), indicating that glucokinase-expressing neurons in the SON are GE in type. The glucokinase-induced release of oxytocin is consistent with the PVN's role in satiety, as PVN oxytocin neurons project to the NTS to induce CCK release (18). However, the role of glucokinase in the PVN has not been greatly examined, and further research is needed to determine whether it is involved in satiety signaling.

Low levels of glucokinase are also expressed in other brain regions. These include the cerebral cortex, cerebellum, lateral habenula, bed nucleus stria terminalis, inferior olive, retrochiasmatic and medial preoptic areas, and the thalamic posterior paraventricular, interpeduncular, oculomotor, and anterior olfactory nuclei (5, 7, 34, 85, 92, 135). Glucokinase expression has also been found in several raphe nuclei in the brainstem, including the raphe obscurus, raphe pallidus, raphe magnus, and raphe pontis (93). However, its role in these neuronal areas is unknown. The low levels of the enzyme suggest that its function may be of lesser importance compared with other regions discussed.

## CLINICAL IMPLICATIONS: CAN TARGETING GLUCOKINASE HELP IN DIABETES AND OBESITY?

### Glucose Homeostasis and Diabetes

The rising prevalence of T2DM, characterized by high plasma glucose levels due to increased glucose production and an impaired response to insulin, is a considerable health and socioeconomic problem prompting the development of new treatments. In 2014, 387 million individuals were affected by diabetes worldwide, a figure expected to nearly double by 2035 (66a). Treatments have focused on the peripheral organs such as the pancreas and liver. Targeting the brain may provide a novel mechanism to stimulate insulin secretion in T2DM patients (119, 156). Glucokinase activity in the hypothalamus may be involved in peripheral insulin secretion, as icv administration of glucokinase inhibitors reduced GSIS in rats (119). Theoretically, augmenting its activity specifically in this region may hence boost insulin secretion from β-cells. The effects of increased glucokinase activity via pharmacological manipulation have been studied in the VMN and ARC (63, 73). Given the close functional relationship between glucokinase and K_ATP_ channels, sulfonylureas may also augment insulin secretion via the brain; however, this remains to be tested.

In rodents, extracellular glucose levels in neuronal glucose-sensing centers such as the VMH typically vary between 0.5 and 2.5 mM and remain ∼0.5 mM in other brain areas (39, 167). The neuroendocrine form of glucokinase is most sensitive to glucose within this concentration window (73). A study in rats has shown that CSF glucose levels do not rise above 4.5 mM (146). Levels of CSF glucose in diabetes are presumed to be higher than in healthy humans, although this has not been properly established; however, a similar limit in glucose transport once a certain threshold has been reached is probable, as humans and rodents have similar glycaemic profiles (169). Therefore, CNS glucose levels in diabetes are likely to be lower than in the periphery.

Hypoglycemia unawareness is a challenge in the management of diabetes (9). It results from abnormalities in glucose-sensing leading to defective CRR to IIH (98). Enhancing the CRR by restoring glucose-sensing pathways may rectify hypoglycemia unawareness by contributing to the prevention of IIH due to insulin treatment. Intracerebroventicular infusion of a low-dose hexokinase inhibitor, glucosamine, boosted feeding responses to glucoprivation in rats with impaired CRR (120). Limitations of this study in applying these findings to glucose sensing and glucokinase were discussed earlier; however, they suggest a potential for glucose-sensing modulation in enhancing orexigenic signaling during hypoglycaemia. A recent approach via a mechanism downstream to glucokinase utilized the K_ATP_ channel activator diazoxide to improve CRR to IIH in humans (54). Targeting glucokinase in the VMH or MAN may provide an alternative strategy to treat this difficult disorder.

### Appetite and Obesity

The recent rise in obesity is a growing concern. The World Health Organization estimates that in 2014 more than 1.9 billion adults were overweight, of which 600 million were obese, fueling the pressing need for treatments (168). Obesity is an important risk factor for cardiovascular diseases and metabolic disorders such as T2DM (65).

We recently provided evidence that ARC glucokinase regulates feeding and preference for glucose-rich foods (63). Low glucose levels, which lead to food-seeking behavior, have been shown to enhance glucokinase expression (75). Glucokinase activation promotes NPY secretion in the ARC, which may drive food intake (63). An inhibitor targeting glucokinase specifically in this region could potentially reduce appetite. Supporting this, the anorexigenic peptide GLP-1-(7–36), which has the opposite effect of NPY on satiety, significantly reduced cerebral glucose metabolism in human hypothalamus and brainstem. GLP-1-(7–36) administration may impair glucose transport by reducing GLUT2 expression and/or glucose phosphorylation by glucokinase. These components are colocalized in hypothalamic neurons, suggesting that a glucose-sensing system may be involved in the transduction of satiety signals (5). The beneficial effects of GLP-1-(7–36) on glucose metabolism support a potential role for ARC glucokinase inhibitors in the regulation of appetite. In vivo studies are required to determine whether such agents can bypass the BBB and act directly in the hypothalamus.

## CONCLUDING REMARKS AND FUTURE PERSPECTIVES

Glucokinase is critical for neuronal glucose sensing and energy homeostasis. Earlier work has demonstrated an important role for this neuronal enzyme in the hypothalamus. More recent studies have supported this but also extended its importance beyond the hypothalamic region. As suggested in this review, there are implications from these studies for the development of effective drugs against the increasingly prevalent obesity and T2DM.

It is important to note that although glucose is an important energy signal, other metabolic signals also play a role in energy homeostasis (87). Insulin can alter neuronal depolarization (152) by acting on K_ATP_ channels (167) or via the insulin-sensitive GLUT4 (74). Hypothalamic glucose-sensing neurons are also sensitive to changes in fatty acid (166), lactate (151), or ketone body (103) levels. It is unclear whether glucokinase plays a role in mediating the response to these various signals.

Research identifying the role of neuronal glucokinase has some limitations. In some instances, manipulating plasma glucose levels may alter glycemia outside physiological levels. The findings thus may not be representative of glucokinase's role in normal conditions. In addition, quantification of glucokinase expression in the brain does not allow measurement of its neuronal activity, and its presence is not necessarily indicative of its involvement in any neuronal processes. Another obstacle is targeting the appropriate brain region with pharmacological agents or viral vectors in vivo. Intranuclear injections require immense precision, and their accuracy often cannot be verified until the end of the study.

Much remains to be learned about the role of neuronal glucokinase. Potential avenues to explore include identifying downstream targets for glucokinase's effect on glucose appetite, obesity, and glucose homeostasis, exploring its role in regions outside the hypothalamus, and characterizing the glucose-brain-islet pathway causing GSIS. Finally, recent works suggest possible targets for diabetes and obesity treatments. They also prompt review of possible off-target effects from glucokinase activators currently in clinical trials that may promote appetite and weight gain. The influence of nonneuronal cells such as tanycytes, not discussed in this review, in glucose sensing requires further characterization. Glucokinase regulatory protein (GKRP) expressed in rat (136) and human (134) brains can interact with glucokinase. Although further investigation is needed to detail the relationship between glucokinase activity, GKRP, and glucose sensing, it raises the potential of influencing glucokinase activity and glucose sensing via alternative mechanisms.

## GRANTS

This article was funded by Biotechnology and Biological Sciences Research Council (BBSRC) project Grant No. BB/I00842X and supported by the National Institute for Health Research (NIHR) at Imperial College Healthcare National Health Service (NHS)Trust. The views expressed are those of the authors and not necessarily those of the BBSRC, the NHS, the NIHR, or the Department of Health. The Section of Endocrinology and Investigative Medicine is funded by grants from the Medical Research Council, BBSRC, and NIHR as well as the NIHR Imperial Biomedical Research Centre Funding Scheme, an Integrative Mammalian Biology Capacity Building Award, and an FP7-HEALTH-2009-241592 EurOCHIP grant. S. S. Hussain was funded by Wellcome Trust Clinical Research Fellowship Grant No. 090792/Z/09/A.

## DISCLOSURES

No conflicts of interest, financial or otherwise, are declared by the authors.

## AUTHOR CONTRIBUTIONS

I.D.B. and S.S.H. conception and design of research; I.D.B. prepared figures; I.D.B. drafted manuscript; I.D.B., S.S.H., S.R.B., and J.V.G. edited and revised manuscript; I.D.B., S.S.H., S.R.B., and J.V.G. approved final version of manuscript.
